# Anti-science kills: From Soviet embrace of pseudoscience to accelerated attacks on US biomedicine

**DOI:** 10.1371/journal.pbio.3001068

**Published:** 2021-01-28

**Authors:** Peter J. Hotez

**Affiliations:** 1 Departments of Pediatrics and Molecular Virology & Microbiology, Texas Children’s Center for Vaccine Development, National School of Tropical Medicine, Baylor College of Medicine, Houston, Texas, United States of America; 2 Hagler Institute for Advanced Study at Texas A&M University, College Station, Texas, United States of America; 3 Department of Biology, Baylor University, Waco, Texas, United States of America; 4 James A Baker III Institute of Public Policy, Rice University, Houston, Texas, United States of America; 5 Scowcroft Institute of International Affairs, Bush School of Government and Public Service, Texas A&M University, College Station, Texas, United States of America

## Abstract

The United States witnessed an unprecedented politicization of biomedical science starting in 2015 that has exploded into a complex, multimodal anti-science empire operating through mass media, political elections, legislation, and even health systems. Anti-science activities now pervade the daily lives of many Americans, and threaten to infect other parts of the world. We can attribute the deaths of tens of thousands of Americans from COVID-19, measles, and other vaccine-preventable diseases to anti-science. The acceleration of anti-science activities demands not only new responses and approaches but also international coordination. Vaccines and other biomedical advances will not be sufficient to halt COVID-19 or future potentially catastrophic illnesses, unless we simultaneously counter anti-science aggression.

## Abstract 2

This Essay argues that COVID-19 exposed how a rising tide of anti-science rhetoric and activities can dramatically exploit society's vulnerabilities to an infectious disease, suggesting that anti-science extremism has become as big a threat as the virus itself.

*“Without science, democracy has no future*.*”—Maxim Gorky, April 1917*

The newest (October 2020) projections from the University of Washington Institute of Health Metrics and Evaluation (IHME) Coronavirus Disease 2019 (COVID-19) forecasting team reveal a grim reality. Their estimates indicate that more than 510,000 Americans could lose their lives by February 28, 2021 [1], representing more than a doubling of the current estimates of 220,000 deaths (although not all groups agree with these estimates). For most of 2020, the US has been the epicenter of the COVID-19 pandemic, leading the world in new cases and deaths. This dire situation is a consequence of our government’s failure to launch a coordinated national response and roadmap and refusing to aggressively promote nonpharmaceutical interventions (NPIs), especially face masks, social distancing mandates, school closures, testing, and contact tracing [2]. In its place, and as the cases and deaths mounted, the White House and its coronavirus task force, and famously the President himself, organized a campaign of disinformation [3].

Central to White House anti-science promotion efforts were attempts by key officials to downplay the severity of COVID-19 and its long-haul consequences, inflate the curative properties of certain medicines such as hydroxychloroquine, falsely attribute COVID-19 deaths to comorbidities in order to artificially reduce actual disease mortality rates, and make scientifically unsubstantiated claims about herd immunity (or its links to the Great Barrington Declaration, which argued without evidence that restrictions cause more harm than the virus). There were also efforts to discredit the effectiveness of face masks to prevent COVID-19 or to refuse implementing mask mandates, invoking at times new political terms or slogans that gained popularity in recent years such as “health freedom” or “medical freedom” [4]. This is exemplified by a recent October 22 tweet from the Republican Governor Kristi Noem of South Dakota [5]:

*If folks want to wear a mask, they are free to do so. Those who don’t want to wear a mask shouldn’t be shamed into it, and govt should not mandate it. We need to respect each other’s decisions. In SD, we know a little common courtesy can go a long way*.

The open questioning of face masks or refusal to enforce mandates will likely continue to have tragic consequences for the American people. According to the IHME COVID-19 forecasting team, 95% public mask use would save almost 130,000 lives from September 22, 2020, through February 28, 2021 [1]. Thus, anti-science disinformation that advocates shunning masks could inflict a mass casualty event in the US. Its occurrence should not surprise us. Instead, our tragic loss of American lives would reflect the handiwork of an evolving anti-science movement that aggressively accelerated in the last 5 years beginning in California and Texas. In this Essay, I argue that to understand how a nation state might seek to attack and dissolve modern biomedicine, it is helpful to revisit a tragic period in 20th century Russia (see Box 1). The relentless attacks on science and scientists during Stalin’s Great Purge and the ascendancy of Lysokenkoism and other pseudoscientific theories provide a useful framework for addressing some stark reminders about the politicization of science occurring now in America, even if they play out at a far lesser scale.

**Box 1. Lessons from a dark chapter in history**

One of the darkest chapters in the history of the Soviet Union, the Great Purge, or the Great Terror (Большой террор), saw the widespread imprisonment, execution, and persecution of millions considered an enemy of Joseph Stalin’s government. It began following the assassination of Sergei Kirov in 1934, a Soviet leader and revolutionary, before halting in 1938, although significant elements of the purge remained throughout the 1940s. The intelligentsia was a Great Purge target, as were entire fields of science, including astrophysics, which was ultimately deemed as a “political platform” running counter to Marxism [6]. Another was the field of mendelian genetics, then led in the USSR by Nikolai Vavilov in his role as head of the Lenin All Union Academy of Agricultural Sciences, the scientific branch of the Commissariat of Agriculture. Vavilov was a botanist and a scientific pioneer in using genetic approaches to improve cereal crops for the USSR [6–8]. Ultimately, Vavilov came under attack by Trofim Lysenko, a peasant with no doctoral degree who popularized and laid claims to the concept of “vernalization” [6]. Lysenko and his colleagues proposed moistening and chilling winter wheat and allowing it to germinate in order to sense these conditions in time for the spring when it would supposedly flourish [6]. Through vernalization—which bore some resemblance to Lamarckian evolutionary theories by claiming that acquired traits could be inherited—Lysenko aspired to adapt wheat to the harsh Russian climate. As a sort of proof of concept, he had his father soak his winter wheat in water before burying it in a snowbank to keep it cold prior to spring planting [6].

Initially, Vavilov took on a mentoring role for Lysenko, even touting his accomplishments at the Sixth International Congress of Genetics held at Cornell University in Ithaca, New York, in the summer of 1932 [8]. See, for example, Vavilov’s praise of Lysenko in a special news “flash,” as it was called by R. C. Cook, the editor of the *Journal of Heredity* during the 1940s [8]:

*The remarkable discovery recently made by T. D. Lysenko of Odessa opens enormous new possibilities to plant breeders and plant geneticists of mastering individual variation.. . .The essence of these methods, which are specific for different plants and different variety groups, consists in the action upon the seeds of definite combinations of darkness (photo-periodism), temperature and humidity. This discovery enables us to utilize in our climate for breeding and genetic work tropical and sub-tropical varieties.... This creates the possibility of widening the scope of breeding. . . to an unprecedented extent, allowing the crossing of varieties requiring entirely different periods of vegetation*.

Lysenko’s vernalization technology would theoretically make it possible, argues Simon Ings in his book, *Stalin and the Scientists*, “to grow alligator pears and Bananas in New York and lemons in New England” [6]. Its extraordinary claims aside, vernalization was seen as a form of Soviet homegrown science and a source of national pride. In contrast, Lysenko was able to convince Stalin that genetics was an evil science, much like relativity. Political expediency became the rationale for promoting pseudoscience even if it meant that millions of rural peasants would die of starvation in the USSR when Lysenko’s cold-resistant crops failed to materialize. Ultimately, Lysenko became the President of the Lenin Academy of Agricultural Science in 1939, whereas Vavilov was arrested in 1940 and rounded up with other intellectuals, including the founder of the Marx-Lenin Institute of World Literature. He was interrogated and sent to a Soviet prison in Saratov where he perished, possibly by starvation in January 1943, despite repeated appeals from international leaders including British Prime Minister, Winston Churchill (Fig 1) [6].

**Fig 1:**
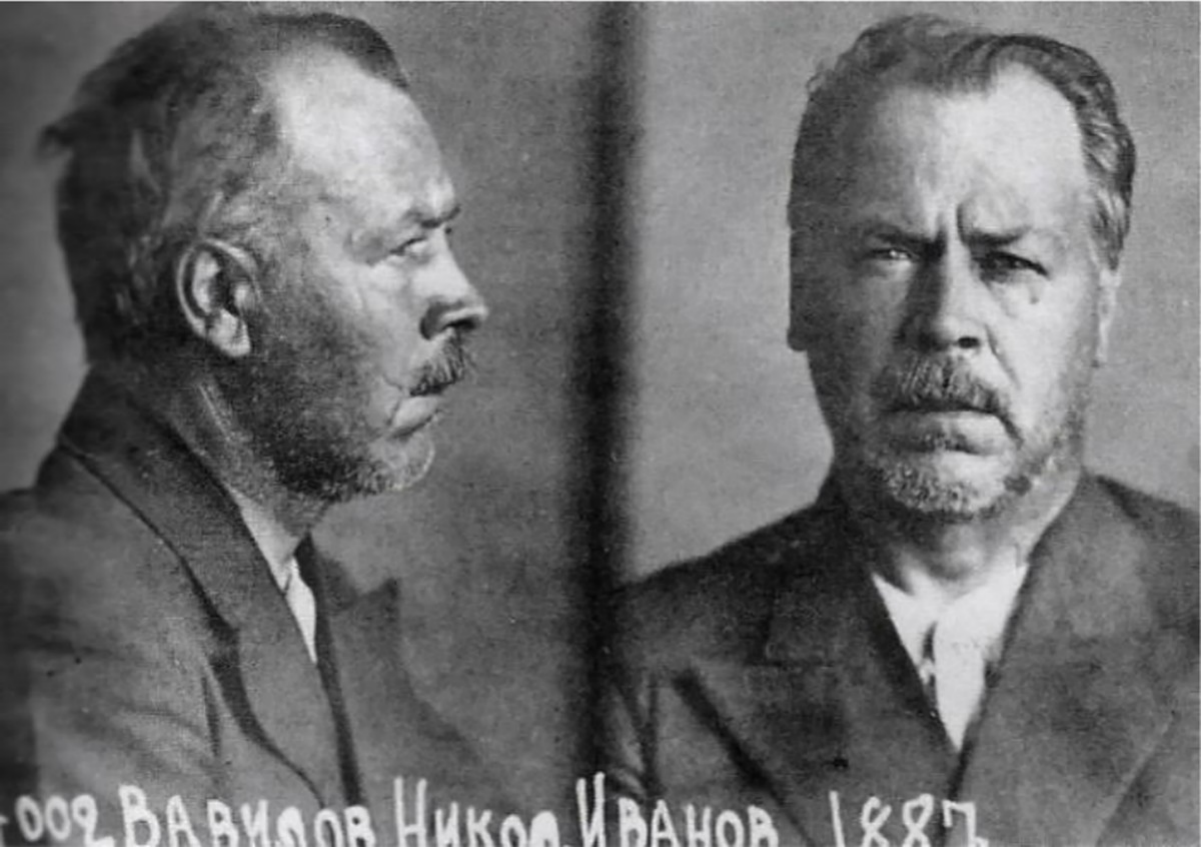
Photo of the prisoner Nikolai Vavilov. Official photo from the file of the investigation. The People's Commissariat for Internal Affairs (Народный комиссариат внутренних дел), Central Archive of the Federal Security Service of the Russian Federation (Moscow) (Центральный архив ФСБ РФ (Москва)) Institute of Plant Industry (Всероссийский институт растениеводства имени Н. И. Вавилова), created January 1, 1942. https://en.wikipedia.org/wiki/Nikolai_Vavilov#/media/File:Vavilov_in_prison.jpg.

Vavilov received a posthumous pardon by Nikita Khrushchev during the 1950s, and in 2008, a book about his life, *The Murder of Nikolai Vavilov*: *The Story of Stalin's Persecution of One of the Great Scientists of the Twentieth Century* was published in English [9]. It remains a great irony that Vavilov devoted his scientific career to the humanitarian cause of feeding the population of the Soviet Union only to die by starvation.

Following the death of Stalin in 1953, the USSR began reopening to international science, ushering in a new era in vaccine development. Throughout the 1950s, both the US and Soviet Union suffered from severe polio epidemics prompting the 2 nations to embark on an unprecedented scientific collaboration [10]. Dr. Albert Sabin sent his polio strains to the USSR where they were manufactured at large scale to produce a trivalent vaccine. During the “Khrushchev Thaw,” it was tested in tens of millions of Soviet citizens and shown to be both safe and effective at preventing polio. A decade later, the US and USSR collaborated to improve a vaccine leading to the eradication of smallpox [10]. Nonetheless, state oppression of Soviet scientists continued, and Krushchev supported Lysenko’s work. Moreover, the physicist and father of the Soviet hydrogen bomb, Andrei Sakharov, won the Nobel Prize in 1975 advocating for human rights, but was subsequently arrested and exiled to Gorky [11]. The mathematician and chess champion, Natan Sharansky, was arrested on treason charges in 1977 and kept in solitary confinement before he was released through a prisoner exchange, later emigrating to Israel in 1980. The American physicist Robert Oppenheimer also endured persecution during the red scare in the 1950s, though on a lesser scale, having had his national security clearance revoked.

## An anti-science legacy through vaccines

Although the exploitation of biomedical anti-science as a political instrument reached its darkest hour during the Great Purge, it did not end with Lysenko. Today, Russian politicization of biomedicine—the biological sciences as they apply to translational medicine—reveals a confusing or ambivalent system of legitimate scientific endeavors alternating with an ever-widening program of disinformation designed to undermine the field. This is especially true in the area of vaccines. For example, the Russian adenovirus-vectored COVID-19 vaccine has shown some promise in published Phase I trials [12], although its rapid registration ahead of Phase III or pivotal studies, together with propaganda calling it “Sputnik V,” raises concerns surrounding its quality control or assurances [13]. An irony is that a parallel and vast Russian anti-vaccine internet campaign is helping to undermine public confidence in vaccines [14], with Sputnik V potentially swept into the disinformation vortex as collateral damage. Such Russian “weaponized health communication” works through computer bots and trolls to create international discord about vaccines [14].

In the US, Russian vaccine disinformation on the internet amplifies an anti-vaccine movement that began following publication of a 1998 paper by Andrew Wakefield and his colleagues alleging links between measles vaccinations and autism [15,16]. However, the damage from Russian bots and trolls accelerated starting around 2014 ahead of the US Presidential election. It coincided with the anti-vaccine movement’s embrace of political extremism, especially on the right [17]. During this time, the anti-vaccine movement began rallying behind medical freedom to counter the introduction of bills in the California legislature designed to close nonmedical exemptions for vaccines. The new laws were disparaged by at least 1 Republican California state senator as “a direct attack on our liberty and a violation of personal rights,” with another legislator asserting “[w]e do not have the right, nor should we have the power, to take away a parent’s right to choose” [18]. In 2015, Texas rapidly adopted and expanded this new reframing of vaccine refusal leading to the formation of Texans for Vaccine Choice and other medical freedom groups [17,19]. The number of children denied access to their vaccines required for school entry increased significantly in Texas and other western states [20,21]. Ultimately, measles outbreaks returned to the US almost 20 years after this childhood infection was eliminated [16].

## Convergence and expansion: Exploiting the COVID-19 crisis

From Texas and California, medical freedom and the anti-vaccine movement spread across the US, running in parallel or linked to a new national expression against vaccines. By 2019, a confederation of anti-vaccine groups occupied almost 500 websites, amplifying on social media and e-commerce and dominating the internet [16]. Russian weaponized health communication continued to sow discord.

When COVID-19 emerged in Europe and North America at the beginning of 2020, many of us in the scientific community were initially optimistic that the anti-vaccine movement might go undercover or disappear from public view. However, the opposite happened as anti-vaccine activists added medical freedom protests against face masks, social distancing, and contact tracing [22,23]. This had devastating consequences as COVID-19 surged in the Southern US during the summer, roughly accounting for at least a third of the more than 220,000 American deaths by October 2020.

Fueling medical freedom ideologies was an active and unabashed anti-science disinformation initiative by the White House that resembled similar approaches from the Brazilian President Jair Bolsonaro and Philippines President Rodrigo Duterte. These government-led anti-science campaigns, since known as “medical populism,” combined multiple strategies: obscuring the gravity of the COVID-19 pandemic, dismissing the severity of COVID-19, hyping miracle cures, and inflating or “spectacularizing” the abilities of Presidents Trump, Bolsonaro, or Duterte to fight the disease [24,25].

Although President Trump did not win reelection and the new Biden administration has vowed to mount an evidence-based pandemic strategy, the anti-science pursuits of the Trump White House and Coronavirus Task Force have caused serious harm. They disparaged prominent US scientists or accused them of political motivations, altered or blocked guidance documents and recommendations from US Department Health and Human Service agencies such as the US Centers for Disease Control and Prevention (CDC) and Food and Drug Administration (FDA), and touted unsubstantiated conspiracy theories about the Chinese Communist Party and the World Health Organization.

The White House Coronavirus Task Force further misled the American public into dismissing social distancing guidelines. They exaggerated claims about the US reaching herd immunity without vaccination or how K-12 schools and colleges could be opened safely even in areas of high virus transmission [26]. The President even asserted that his Democratic opposition in the 2020 election was “antivaccine” due to its support for reinforced FDA guidance to ensure that Operation Warp Speed vaccines released through emergency use authorizations are adequately tested for safety and efficacy. Currently, several efforts are now underway to provide a full accounting of the White House anti-science activities, including those that expand beyond biomedicine [27–29].

During the summer and fall of 2020, the US campaign against evidence-based biomedicine spread into Europe. Medical freedom protests against masks, vaccines, contact tracing, and other COVID-19 preventive measures were held in multiple European capitals this fall, including Berlin, Germany, London, England, and Paris, France [23]. Of particular concern were news reports linking these protests to far-right extremist political activities [23]. Therefore, what began as a homegrown medical freedom protest against vaccines expanded into other aspects of biomedicine with connections to far-right extremist activities in America, and ultimately globalized through Russian cyberattacks, and ominous and politically affiliated protests in Western Europe [23].

## The fix

These developments suggest that an anti-science confederacy or empire may be on the verge of expanding across the Northern Hemisphere. A triumvirate of (1) medical freedom initiatives in the US, (2) Russian disinformation, and (3) far right wing extremist groups in Western Europe, now accelerates mistrust in vaccines and COVID-19 prevention [23]. By halting vaccination and prevention programs, it may have already led to tens of thousands of deaths in the US and other group of 20 (G20) nations [30].

Beyond promoting accurate information about vaccines and COVID-19 prevention, we must also consider steps to dismantle the accelerating disinformation. In other words, relying exclusively on fine-tuning or reinforcing pro-vaccine or pro-science messages may no longer suffice. Our messages too often are messages in bottles floating in an ocean of disinformation. We need to address the ocean. We must recognize the potential necessity of assertively confronting anti-science, even if this exceeds the usual confines of biomedicine or the comfort zone of scientists. This may include an appetite to dismantle and remove anti-science content and organizations from the social media and e-commerce sites.

In the US, deconstructing anti-science would require shaping an interagency government task force, which might include representation from the major US Department of Health and Human Services (e.g., CDC, FDA, or US National Institutes of Health (NIH)), but also the Departments of Justice, State, Commerce, and Homeland Security. The inter-agency task force could also recommend a comprehensive initiative to fight anti-science through implementing relevant programs of the US NIH or National Science Foundation (NSF). It might include doctoral and postdoctoral education program for science engagement and communication not currently in place in most US research universities [21]. Potentially, such efforts might include combating creationism or other forms of pseudoscience as they become increasingly mainstreamed (and identified as real science) in the US. In this respect, there are several efforts to introduce legislation on this front in several states [31]

Globally, through the 2020 United Nations (UN) General Assembly, WHO and other UN agencies launched plans to fight a growing “infodemic” through the dissemination of accurate and evidence-based scientific information, and actively countering the disinformation empire “while respecting freedom of expression” [32]. However, details of such efforts and the extent to which UN agencies are willing to carry this fight remain unknown. Other international bodies might offer assistance including the North Atlantic Treaty Organization’s (NATO’s) Science for Peace and Security Programme [33].

It remains unclear whether the UN agencies or NATO are resourced adequately to implement an effective counterpunch against anti-science disinformation. For example, fully combating anti-science may require far-reaching measures that include taking on larger and better-resourced organizations. Ironically, they include tech giants as ubiquitous sources of anti-science disinformation, especially in regards to vaccines [34]. Even large communications conglomerates, such as News Corp and its sister organization, have come under fire for promoting White House COVID-19 disinformation during the 2020 US Presidential campaign [35,36]. James Murdoch, who resigned from the board of News Corp over editorial differences, criticized such actions [35]:

*A contest of ideas shouldn’t be used to legitimize disinformation. And I think it’s often taken advantage of. And I think at great news organizations, the mission really should be to introduce fact to disperse doubt—not to sow doubt, to obscure fact, if you will*.

Government-led anti-science disinformation also remains potent. According to The New York Times, one of Russia’s major news organizations, originally known as Russia Today but since named RT, (and its US arm known as RT America) comprises a key element for a vast “ecosystem” of anti-science disinformation targeting COVID-19, according to the Global Engagement Center of the US Department of State [37]. Increasingly, these activities are being tied closely to both the Kremlin and Russian President, Vladimir Putin [37].

Beyond the UN agencies, the leaders of the G20 nations will also be required to expand counter measures targeting anti-science at the international level. A reality is that the overall budgets of our major science-based UN organizations pale in comparison to for-profit enterprises highlighted above.

Despite such vast financial differentials, we must begin coordinating an alliance committed science organizations to combat the anti-science empire. We might host a major summit on this topic at the next UN General Assembly or G20 summit and begin implementing programs of public awareness to highlight the public health consequences arising from a global attack on biomedicine. In parallel, we must begin efforts for strategic planning and engagement to oppose these new threats to biomedicine. The G20 nations are in a unique position to pressure Russia and the Kremlin to halt their anti-science disinformation efforts.

## Concluding statement

The devastating COVID-19 pandemic of 2020 will soon exceed 100 million global cases and 2 million deaths. We must recognize how the virus had considerable help launching a broadside attack on our global health. An anti-science disinformation campaign of unprecedented magnitude and led by both multinational corporations and some governments, especially the Russian and US Governments, fuels the pandemic. It represents a legacy going back to Russia since the Great Purge of the 1930s to 1940s [38], unraveling great accomplishments in biomedicine that include Russia’s only 2 Nobel Prizes in Physiology or Medicine awarded during the first decade of the 1900s [39]. There is now urgency to develop an array of COVID-19 vaccines and other biomedical interventions. But ultimately, solutions through biomedicine won’t be sufficient to halt the spread of COVID-19. We must simultaneously dismantle anti-science.

## Abbreviations

CDC: Centers for Disease Control and Prevention
COVID-19: Coronavirus Disease 2019
FDA: Food and Drug Administration
G20: group of 20 nations
IHME: Institute of Health Metrics and Evaluation
NATO: North Atlantic Treaty Organization
NPI: nonpharmaceutical intervention
NSF: National Science Foundation
UN: United Nations
